# Community Structure of Protease-Producing Bacteria Cultivated From Aquaculture Systems: Potential Impact of a Tropical Environment

**DOI:** 10.3389/fmicb.2021.638129

**Published:** 2021-02-04

**Authors:** Yali Wei, Jun Bu, Hao Long, Xiang Zhang, Xiaoni Cai, Aiyou Huang, Wei Ren, Zhenyu Xie

**Affiliations:** ^1^State Key Laboratory of Marine Resource Utilization in the South China Sea, Hainan University, Haikou, China; ^2^Ministry of Education Key Laboratory of Cell Activities and Stress Adaptations, School of Life Sciences, Lanzhou University, Lanzhou, China; ^3^Hainan Provincial Key Laboratory for Tropical Hydrobiology and Biotechnology, Hainan University, Haikou, China; ^4^College of Marine Sciences, Hainan University, Haikou, China

**Keywords:** protease-producing bacteria, *Vibrio owensii*, tropical aquaculture, enterobacterial repetitive intergenic consensus-polymerase chain reaction, environmental variables, *Bacillus hwajinpoensis*, bacterial community structure

## Abstract

Protease-producing bacteria play vital roles in degrading organic matter of aquaculture system, while the knowledge of diversity and bacterial community structure of protease-producing bacteria is limited in this system, especially in the tropical region. Herein, 1,179 cultivable protease-producing bacterial strains that belonged to Actinobacteria, Firmicutes, and Proteobacteria were isolated from tropical aquaculture systems, of which the most abundant genus was *Bacillus*, followed by *Vibrio*. The diversity and relative abundance of protease-producing bacteria in sediment were generally higher than those in water. Twenty-one genera from sediment and 16 genera from water were identified, of which *Bacillus* dominated by *Bacillus hwajinpoensis* in both and *Vibrio* dominated by *Vibrio owensii* in water were the dominant genera. The unique genera in sediment or water accounted for tiny percentage may play important roles in the stability of community structure. Eighty *V. owensii* isolates were clustered into four clusters (ET-1–ET-4) at 58% of similarity by ERIC-PCR (enterobacterial repetitive intergenic consensus-polymerase chain reaction), which was identified as a novel branch of *V. owensii*. Additionally, *V. owensii* strains belonged to ET-3 and ET-4 were detected in most aquaculture ponds without outbreak of epidemics, indicating that these protease-producing bacteria may be used as potential beneficial bacteria for wastewater purification. Environmental variables played important roles in shaping protease-producing bacterial diversity and community structure in aquaculture systems. In sediment, dissolved oxygen (DO), chemical oxygen demand (COD), and salinity as the main factors positively affected the distributions of dominant genus (*Vibrio*) and unique genera (*Planococcus* and *Psychrobacter*), whereas temperature negatively affected that of *Bacillus* (except *B. hwajinpoensis*). In water, *Alteromonas* as unique genus and *Photobacterium* were negatively affected by NO_3_^−^-N and NO_2_^−^-N, respectively, whereas pH as the main factor positively affected the distribution of *Photobacterium*. These findings will lay a foundation for the development of protease-producing bacterial agents for wastewater purification and the construction of an environment-friendly tropical aquaculture model.

**Graphical Abstract:**
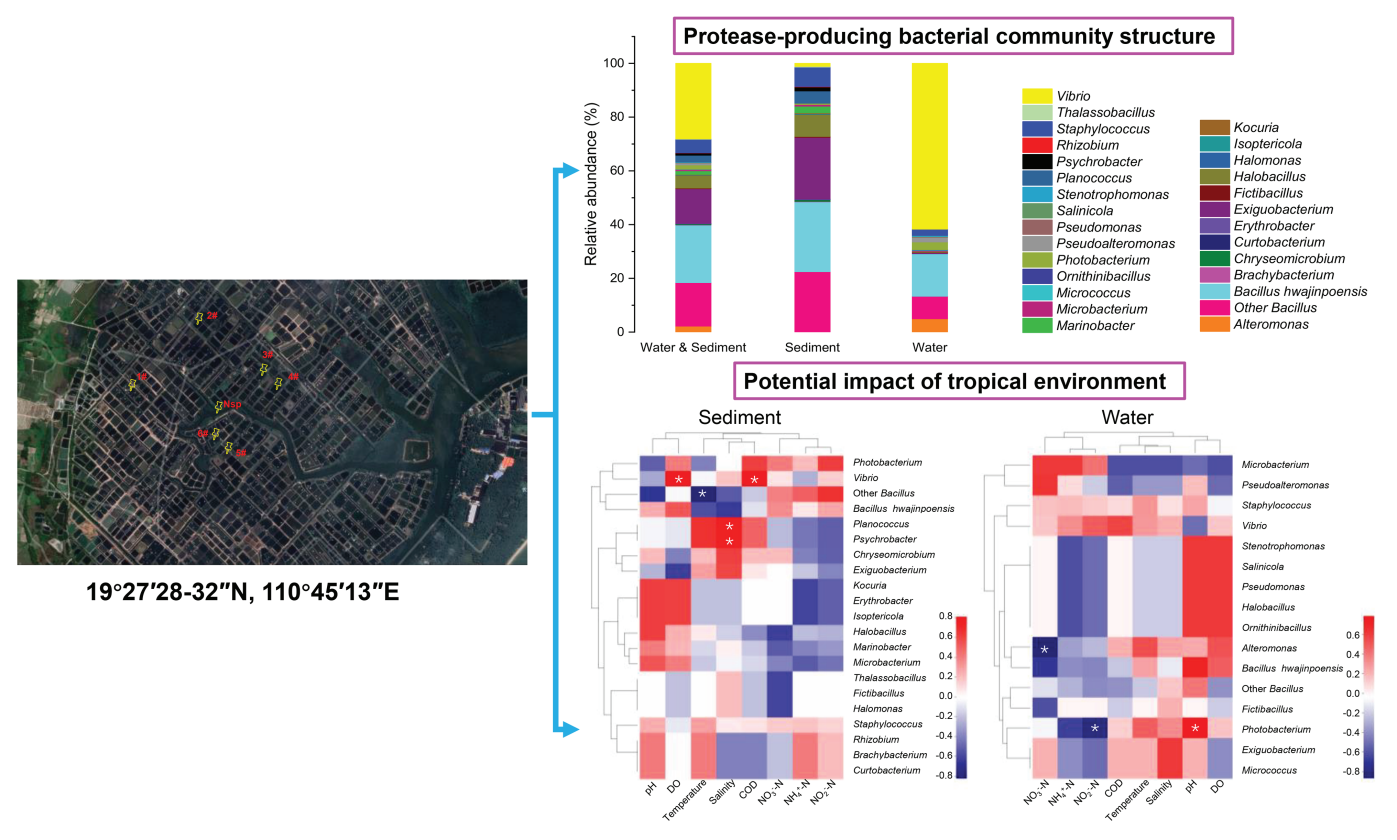
Community structure of protease-producing bacteria cultivated from aquaculture systems: potential impact of a tropical environment, in the case of Wenchang, Hainan, China.

## Introduction

Aquaculture as an important food production has become an important economic activity in many countries (Hamza et al., 2017; Santos and Ramos, 2018). However, with the rapid development of aquaculture industry, a large number of animal residues, residual feed, and excrement have been produced, thereby aggravating the accumulation of organic matter in aquaculture (Lin and Chen, 2001, 2003; Kuhn et al., 2010; Guo et al., 2016; Li et al., 2020; Mariane de Morais et al., 2020). The main sources of nitrogen and carbon of aquaculture are from the particulate organic matters containing large amounts of proteins and amino acids. Generally, particulate organic matter is difficult to be dissolved, which should be first decomposed into dissolved form. In turn, the dissolved organic matter can be transformed into nitrogen gas by ammonification, nitrification, and denitrification. Bacteria play key roles in these processes by secreting degradation enzymes (Olson and Lesser, 2013; Su et al., 2020).

Protease-producing bacteria as the main degraders of organic matter can effectively hydrolyze the organic matter into peptides and amino acids by secreting extracellular proteases (Su et al., 2020). As we all know, peptides and amino acids are essential for subsequent catabolism of organisms. Reportedly, protease-producing bacteria could not only improve protein digestibility and growth of the host, but also reduce organic pollutants in aquaculture (Shi et al., 2016; Amin, 2018; Su et al., 2020). Protease-producing bacteria that belong to four major phyla, Proteobacteria, Firmicutes, Actinobacteria, and Bacteroidetes, have been identified, which are dominated by *Bacillus*, *Pseudomonas*, and *Pseudoalteromonas* genera (Su et al., 2020).

Many methods have been developed to enhance protein digestibility of aquafeed in aquaculture. However, the application of exogenous enzymes has been limited because of expensive and poor stability in various processing conditions. During the feed processing, dietary enzymes may lose their activities under high temperature and pressure conditions. Fortunately, an increasingly accepted method by supplementing protease-producing bacteria considered as an environment-friendly method was good for growth enhancement (Shi et al., 2016; Amin, 2018), which has been confirmed to not only enhance protein digestibility, but also reduce organic pollutants produced by undigested feed (Suzer et al., 2008; Ambas et al., 2015; Reda and Selim, 2015). Reportedly, feed by supplementing 1.0 × 10^10^CFU/g protease-producing bacteria could improve growth and nonspecific immune system of Nile tilapia (Selim and Reda, 2015).

Protease-producing bacteria play a vital role in degrading organic matter. However, the diversity and community of protease-producing bacteria have seldom been addressed in aquaculture. As a consequence, in this article, 1,179 cultivable protease-producing bacterial strains were isolated and screened from sediment and water samples in Wenchang (Hainan, China), and then their diversity and community structure were investigated. Additionally, the physicochemical factors related to diversity and distribution of protease-producing bacterial communities in sediment and water were comprehensively analyzed, respectively. The dominant protease-producing *Vibrio owensii* strains in aquaculture water were classified using enterobacterial repetitive intergenic consensus-polymerase chain reaction (ERIC-PCR).

## Materials and Methods

### Study Site Description and Sample Collection

Surface sediment (2- to 3-cm thickness) and aquaculture water (1-m water depth) samples were collected from six prawn ponds (19°27'28-32''N, 110°45'13''E) and one nature sea pool (closed to the prawn ponds) located at Huiwen Town, Wenchang City, China. The special geographical position is shown in Supplementary Figure S1. The experimental ponds are summarized in Supplementary Table S1.

### Isolation and Screening of Protease-Producing Bacteria

Sediment (0.5g) and water (100μl) samples were serially diluted with sterile normal saline solution (0.85% NaCl) within 24h of collection to obtain 1:10, 1:100, and 1:1,000 dilutions. One hundred microliters of each diluted sample was spread-plated on marine 2216E agar and incubated at 30°C for 18h, which was finally ascertained by preliminary selection for the condition of cultural bacteria. For screening of protease-producing bacteria, 2μl of overnight growth culture in the marine 2216E of each bacterial isolate was spot plated on casein-gelatin agar [containing 0.2% yeast extract, 0.3% xasein, sodium salt, 5% gelatin, 0.13% (NH_4_)_2_SO_4_, 0.05% MgSO_4_·7H_2_O, and 1.5% agar and seawater; pH 7.5–8.0]. The protease-producing bacterial isolates were further used for DNA extraction.

### Quantification of Protease Activity

Protease activity was elevated in terms of hydrolytic capacity (HC); the pure bacterial isolates were cultured in 2166E broth. A solution with OD at 600 nm of 0.2ml was set, of which 1μl was pipetted out and dropped on casein-gelatin agar plate in duplicates. Diameters of bacterial colonies and related-clearance zones were measured after 24-h incubation at 28°C ± 2°C. The HC value determined the protease activity was calculated from the ratio between the diameter of the caseinolytic zone and the diameter of the bacterial colony. In this study, we selected only colonies with a ratio greater than three.

### DNA Extraction and PCR Amplification

Bacterial strain with the highest protease activity was identified using genotypic assay. Genomic DNAs of culturable protease-producing bacteria were extracted using a commercial DNA extraction kit (Tiangen Biotech, Beijing, China; Sun et al., 2008; Marquez-Santacruz et al., 2010), after bacterial growth on 2216E medium and incubation at 30°C for 18h. The 16S rDNA genes were amplified from genomic DNA by PCR using the universal primers 27F (5'-AGAGTTTGATCCTGGCTCAG-3') and 1492R (5'-GGCTACCTTGTTACGACTT-3'). PCR amplifications were performed using the following conditions: initial denaturation of template DNA (95°C for 2 min), then 1cycle consisting of denaturation (30 s at 95°C), annealing (30 s at 60°C), extension (1min at 72°C), 25cycles, and a final extension at 72°C for 5 min. Bacteria sequences obtained by culture-dependent approach were compared with 16S rDNA reference gene sequences by BLAST.^1^

### Genetic Diversity of Dominant *V. owensii* Using ERIC-PCR

All the dominant isolates identified and confirmed as *V. owensii* were chosen for further molecular typing by ERIC-PCR. One microliter of the extracted DNA was added to 12.5μl of PCR amplification mixture, containing 9.5μl ddH_2_O, and 2μl primers (forward and reverse). Amplification reactions were carried out in a thermal cycler (Bio-Rad, United States) using the primer set ERIC-1R 5'-AAGTAAGTGACTGGGGTGAGCG-3', and ERIC-2F 5'-ATGTAAGCTCCTGGGGATTCAC-3'. The amplification program was as follows: an initial denaturation step at 95°C for 7min, denaturation 90°C for 30min; annealing at 52°C for 1min; extension at 65°C for 8min; and after 35cycles, a final extension at 65°C for 16min. Six microliters of each amplified product was electrophoresed on 1% agarose gel with DNA safe stain (Greenview Plus, Andy Gold™, United States) 1 × TAE buffer along with 1 Kb DNA Ladder (Thermo Fischer Scientific, United States) and M2-DL2000 marker (Takara, Japan), at 180V for 50min. The amplification was repeated three times.

Gels were viewed by Gel Imaging System (Tanon3500R, Shanghai, China). The images were captured for further analysis. In turn, “1” and “0” were assigned for the presence or lack of the banding pattern and recorded in sequence according to the reading direction. The genetic patterns were calculated according to the molecular weights and quantification of the bands in each sample. Simpson’s Index of Diversity was used to calculated the discriminatory index of ERIC-PCR (Pishbin and Mehrabian, 2020), and the “unweighted pair group method with dice similarity coefficient” option in NTSYS v. 12 program (Bakhshi et al., 2018) was used to cluster the *V. owensii* isolates.

## Results

### Analysis of Diversity and Distribution of Cultivable Protease-Producing Bacterial Strains

**Figure 1:**
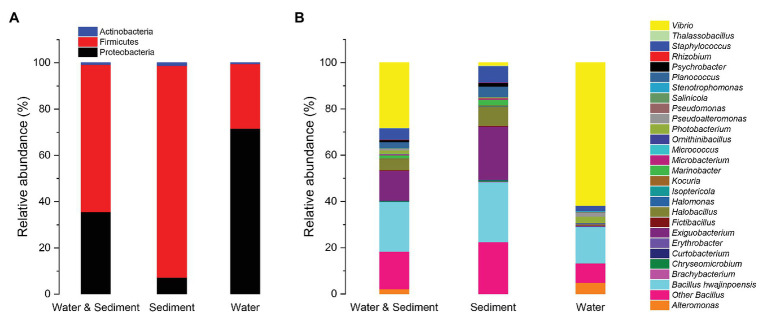
Relative abundances of cultivable protease-producing bacterial strains in water and sediment, sediment, and water of aquaculture systems at the level of phylum **(A)** and genus **(B)**, respectively.

There were 1,179 cultivable protease-producing bacterial strains isolated and screened from sediment (654 strains) and water (525 strains) samples of aquaculture systems, and the bacterial counts of sediment and water varied from 2 × 10^4^ to 2.6 × 10^5^ CFU/g and 658 to 2.1 × 10^3^CFU/ml, respectively (Table 1). All of the isolates belonged to three phyla, Actinobacteria, Firmicutes, and Proteobacteria, and classified into 27 genera dominated by *Bacillus* (37.7%) and *Vibrio* (28.1%) genera (Figure 1, “Water & Sediment” bars), of which Firmicutes accounted for the highest proportion. Additionally, *Bacillus hwajinpoensis* in *Bacillus* was dominant species in whole tropical aquaculture system (Figure 1B). Twenty-one genera from sediment belonged to Actinobacteria, Firmicutes, and Proteobacteria, and bacterial diversity was found to be maximum (up to about 91.6%) in the phylum of Firmicutes. The largest genus was *Bacillus* (*B. hwajinpoensis* as the dominant species, 26%), followed by *Exiguobacterium* in sediment (Figure 1, “Sediment” bars). Additionally, 12 unique genera were isolated only from sediment samples, namely *Brachybacterium*, *Chryseomicrobium*, *Curtobacterium*, *Erythrobacter*, *Halomonas*, *Isoptericola*, *Kocuria*, *Marinobacter*, *Planococcus*, *Psychrobacter*, *Rhizobium*, and *Thalassobacillus*. Sixteen genera from aquaculture water samples also belonged to Actinobacteria, Firmicutes, and Proteobacteria, whereas their relative abundance displayed significant differences with those in sediment samples. The relative abundance of Proteobacteria was up to about 71.6%, followed by Firmicutes (28.0%), and the largest genus was *Vibrio* (61.7%) as the dominant group in aquaculture water samples (Figure 1, “Water” bars). Meanwhile, there were seven unique genera from water samples, namely, *Alteromonas*, *Micrococcus*, *Ornithinibacillus*, *Pseudoalteromonas*, *Pseudomonas*, *Salinicola*, and *Stenotrophomonas*. Previous studies have reported that protease-producing bacteria were extremely diverse, including main genera such as *Acinetobacter*, *Aeromonas*, *Enterobacter*, *Enterococcus*, *Microbacterium*, *Micrococcus*, *Pseudomonas*, *Staphylococcus*, *Stenotrophomonas*, and *Streptomyces*, as well as other unclassified bacteria especially *Bacillus* and *Vibrio*, which were similar to our results (Dat et al., 2019). In this work, 80 *V. owensii* strains among the dominant *Vibrio* genus were isolated. It is worth noting that cultivable protease-producing bacterial diversity of sediment was generally higher than that of water (Figure 1B and Table 1).

**Table 1:** Distribution of cultivable protease-producing bacteria in tropical aquaculture systems.

Ponds	Sediment		Water	
	×10^3^CFU/g	Isolates	×10^3^CFU/ml	Isolates
1#	234.0	148	1.090	79
2#	44.7	62	0.728	93
3#	104.7	54	1.050	105
4#	20.5	148	1.450	45
5#	285.0	132	0.658	76
6#	88.3	75	2.10	113
Nsp	260.0	35	1.820	14
Total		654		525

The aquaculture ponds are coded 1#–6#, and Nsp represents nature seawater pool.

**Figure 2:**
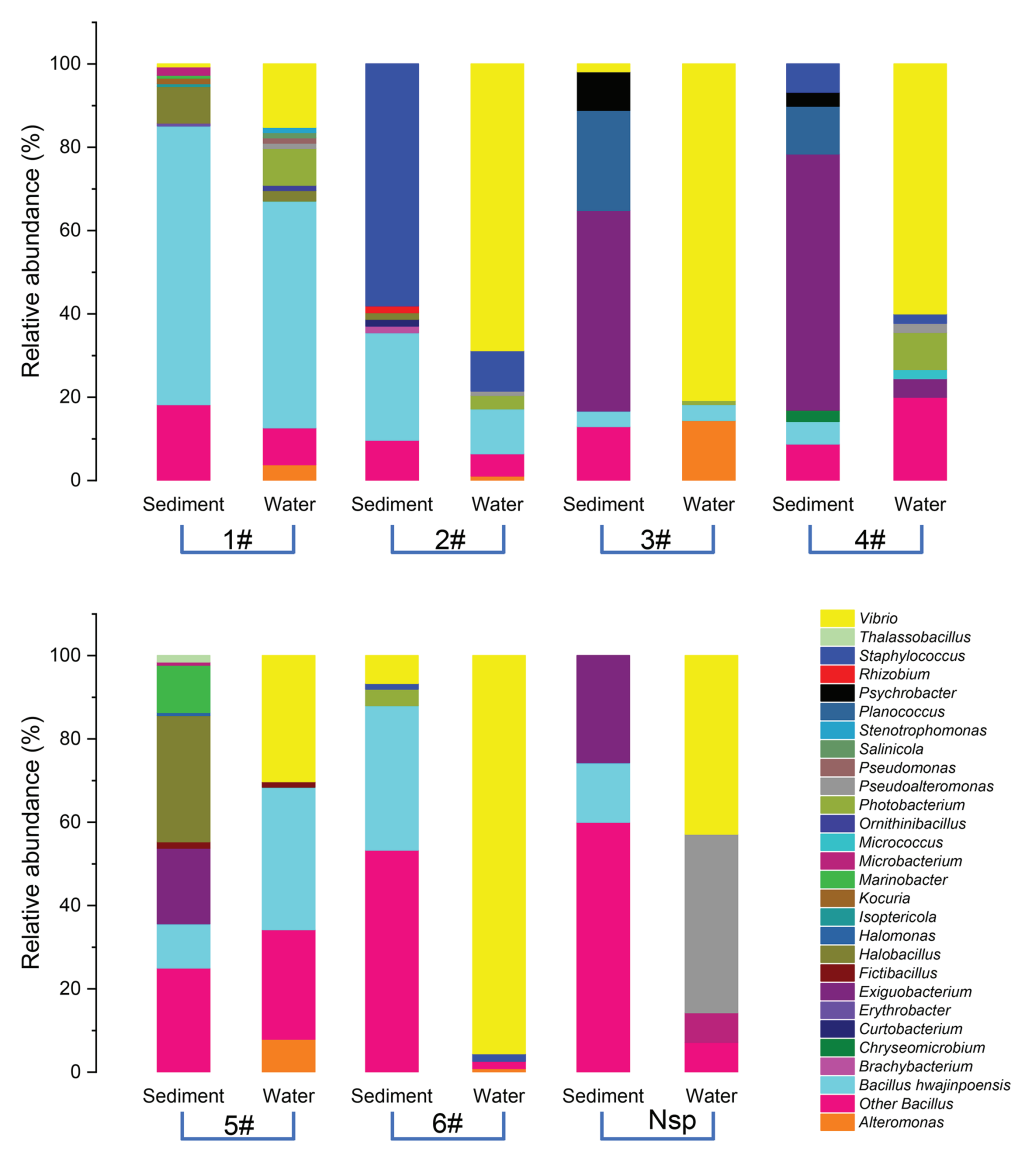
Relative abundances of cultivable protease-producing bacterial strains in sediment and water of aquaculture ponds on the level of genus. The aquaculture ponds are coded 1#–6#, and Nsp represents nature seawater pool.

The diversity and distribution of cultivable protease-producing bacterial strains in different sampling stations were investigated (Figure 2). The results showed obviously different protease-producing bacterial community structures displayed between each aquaculture pond at the genus level. Interestingly, *Vibrio* from water samples was found in all sampling stations (Supplementary Table S2) and accounted for the relatively higher proportion (Figure 2). *Bacillus* also displayed high abundance in most sampling stations.

### Comparison of Cultivable Protease-Producing Bacterial Distribution of Sediments and Water in Aquaculture Systems

**Figure 3:**
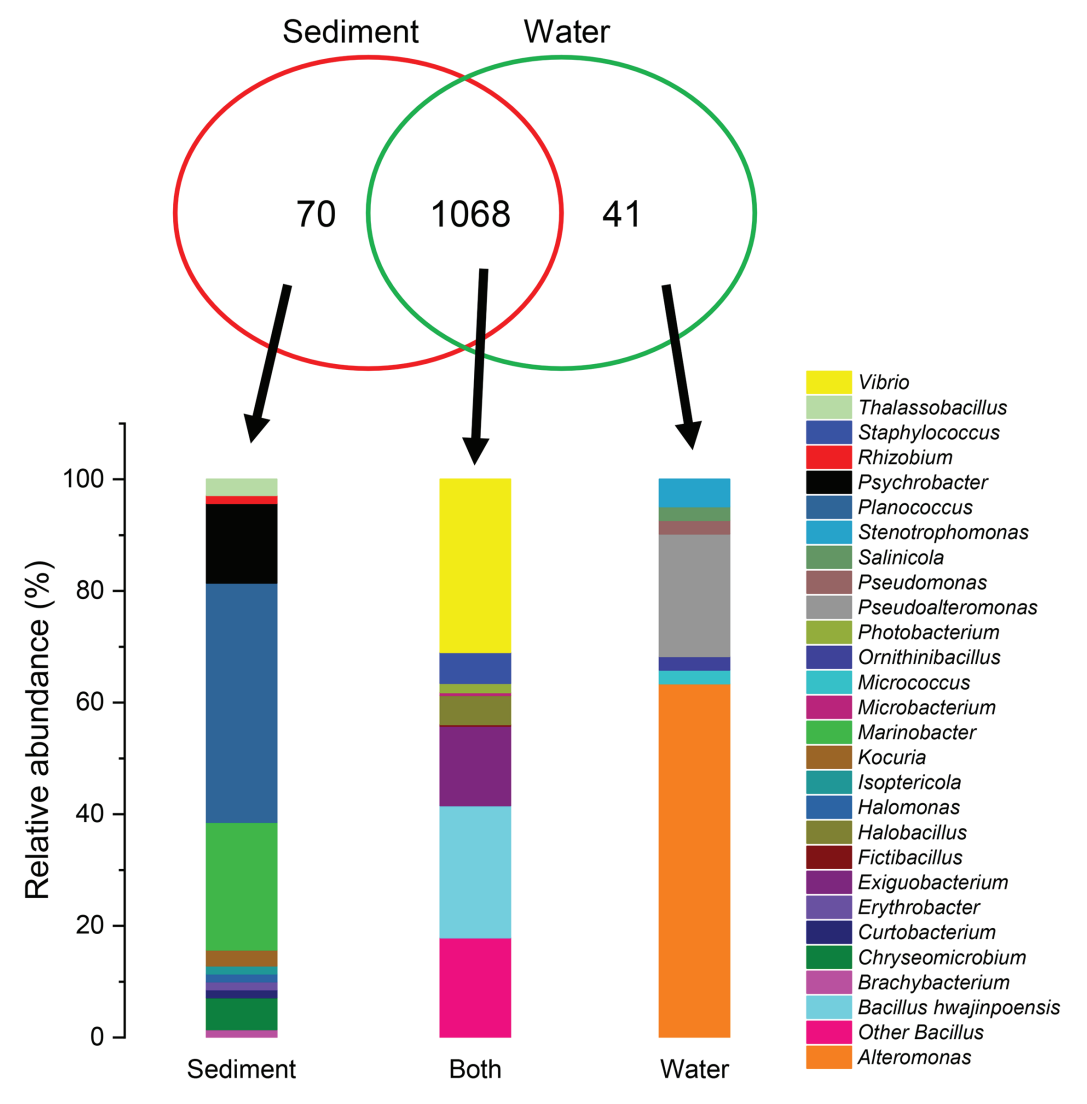
Comparison of the cultivable protease-producing bacteria distribution of sediment and water samples at the genus level. Proportional Venn diagram showing the number of strains found only in sediment samples, only in water samples, and in both. These strains were used to create the relative abundance bar graphs at the genus level under the panel Venn.

As shown in Figure 3, of the 1,179 total cultivable protease-producing bacterial strains from different genera, most strains were found in both sediment and water samples, whereas the sediment and water samples harbored only 70 and 41 unique strains, respectively. *Planococcus* (42.9%) and *Marinobacter* (22.9%) were notably abundant in the unique genus of sediment samples, whereas *Alteromonas* (63.4%) displayed the highest relative abundance in the unique genus of water samples. Among the genera in both locations, *Vibrio* (31.0%) as the dominant group accounted for the highest proportion.

### Genetic Diversity of Dominant *V. owensii* Using ERIC-PCR

**Figure 4:**
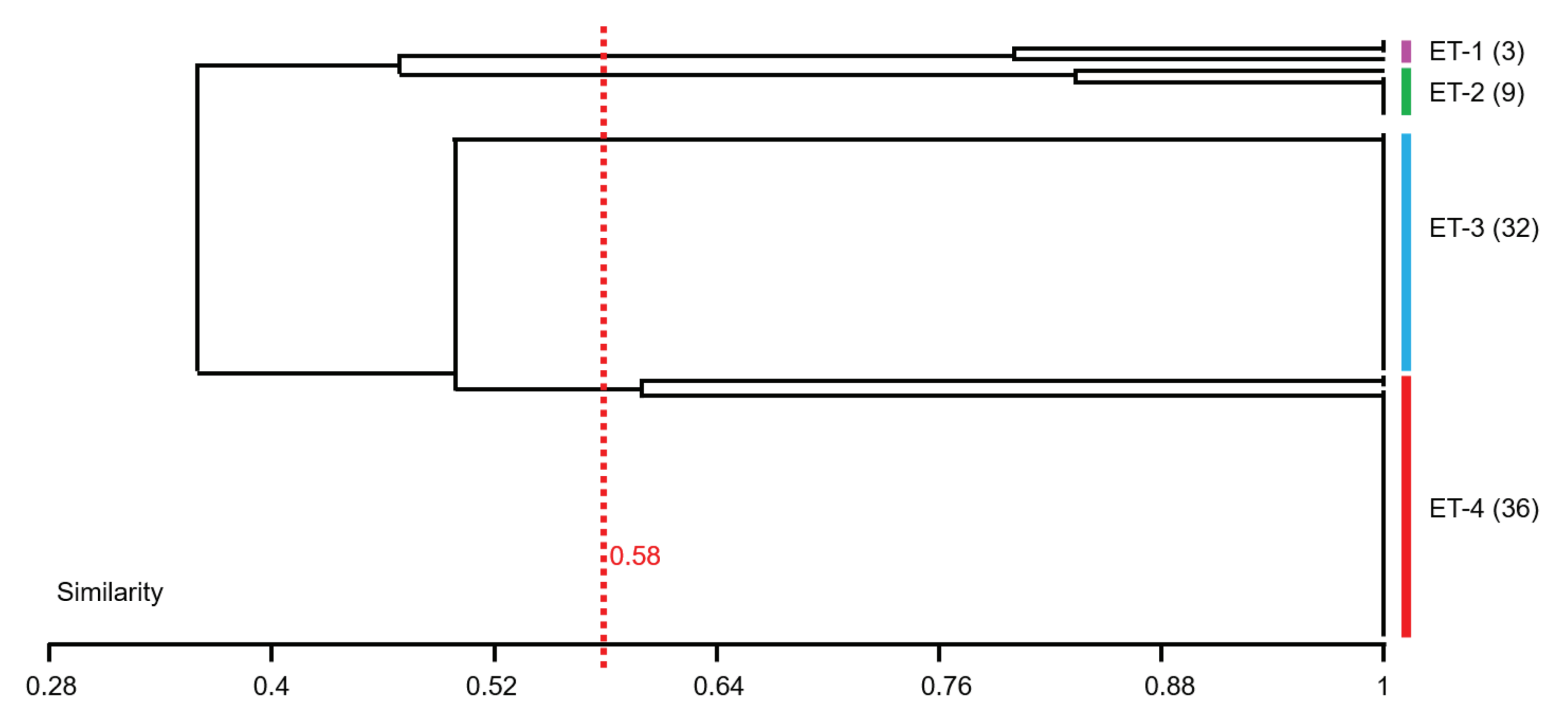
Dendrogram obtained from ERIC-PCR fingerprinting of 80 *Vibrio owensii* strains. Adopting 58% of similarity, isolates were clustered into four clusters, ET-1, ET-2, ET-3, and ET-4.

In this study, 80 *V. owensii* strains as the dominant cultivable protease-producing bacteria were isolated from aquaculture systems. In view of the high-importance *Vibrio* and their increased prevalence in aquaculture, the genetic linkage of these isolates was investigated by ERIC-PCR. In the extragenic regions of *V. owensii* genome, the sequences of enterobacterial repetitive intergenic consensus consist of highly conserved central inverted repeats. In this study, 80*V. owensii* isolates were fingerprinted by ERIC-PCR. This technique generated two to six amplification bands ranging in size from 800 to 7,500bp (Supplementary Figure S2). Adopting 58% of similarity, 80 *V. owensii* isolates were clustered into four clusters (Figure 4 and Supplementary Table S3), ET-1, ET-2, ET-3, and ET-4. The predominant clusters were ET-3 and ET-4, as most isolates had a common banding pattern (7,000bp; Supplementary Figure S2). Only three strains were clustered into ET-1, and nine strains were clustered into ET-2. The Simpson’s Index of Diversity was used to elevate the discriminatory power of this typing method.

### Overview of Physicochemical Parameters in the Survey Area

Temperature varied among different sampling aquaculture ponds, ranging from 28.1 to 29.7°C. pH was relatively stable and varied slightly among the different sampling ponds. Salinity ranged from 20.29‰ to 29.98‰. The differences in dissolved inorganic N, such as NH_4_^+^-N, NO_2_^—^N, and NO_3_^−^-N, were obvious. Physical-chemical parameters of the survey areas were summarized in Table 2.

**Table 2:** Physical-chemical parameters of sampling stations.

Ponds	Temperature (°C)	Salinity (%)	pH	DO (mg/L)	NH_4_^+^-N (mg/L)	NO_2_^−^-N (mg/L)	NO_3_^−^-N (mg/L)	COD (mg/L)
1#	28.9	25.68	7.77	3.12	0.27	0.91	0.025	129.6
2#	29.5	21.87	7.66	2.82	3.01	0.74	0.074	74.8
3#	29.7	26.58	7.41	2.95	1.27	0.25	0.027	141.0
4#	29.4	29.98	7.56	2.42	0.92	0.91	0.025	140.0
5#	29.2	26.30	7.49	2.61	2.20	0.20	0.073	82.0
6#	28.8	26.00	7.28	3.01	2.36	0.99	0.450	147.0
Nsp	28.1	20.29	7.28	1.42	4.03	6.18	0.130	38.8

The aquaculture ponds are coded 1#–6#, and Nsp represents nature seawater pool.

## Discussion

With the rapid development of aquaculture industry, aquaculture system is loaded with abundant organic matter, ammonia-nitrogen, and phosphorus due to high concentration of organic and inorganic pollutants from uneaten feed and aquaculture animal excreta (Dat et al., 2019), and long-term use of antibiotics and chemicals to prevent diseases in aquaculture has led to a series of environmental problems, such as water eutrophication and atmospheric pollution (caused by volatilization of ammonia and hydrogen sulfide; Hai, 2015), which greatly hinders the sustainable development of aquaculture industry. Therefore, exploring a harmless and recyclable technology for reducing excessive organic matter in aquaculture system is necessary to ensure the sustainable development of aquaculture and for environmental protection. Microorganisms such as potential probiotic biocontrol candidates play important roles in diseases resistance and improvement of water quality (Blancheton et al., 2013; Hucheng et al., 2020). Abundant enzymes produced by microorganisms, such as protease, lipase, and amylase, can effectively decompose the excessive bait and other organic matter, which play a key role in material transformation and energy metabolism in aquaculture system. Intensive aquaculture system is a closed or semiclosed material circulation system. Protein and other high-molecular substances from bait are the main sources of pollution in aquaculture system. Therefore, aquaculture system is highly dependent on functional bacteria that can promote the decomposition of high-molecular substances, such as protease-producing bacteria. Meanwhile, tropical marine region with unique climate and environmental condition harbors diverse bacterial communities with unique metabolic and physiological capabilities (Hamza et al., 2017; Ren et al., 2018, 2019). Wenchang in Hainan Island belonging to a tropical marine region is one of the largest aquaculture production areas in China, but the knowledge of protease-producing bacterial community structure in this area, especially in aquaculture system, is extremely limited.

For this purpose, we herein communicate that protease-producing bacteria displayed high diversity and complex community structure. The diversity and relative abundance of protease-producing bacteria in aquaculture sediments were generally higher than those in aquaculture water. Similar results were also found in Guangdong (China) aquaculture system (Wenguang et al., 2015). All of the protease-producing bacteria isolates from the survey aquaculture systems belonged to three phyla, Actinobacteria, Firmicutes, and Proteobacteria, of which Firmicutes accounted for the highest proportion, and the dominant genera were *Bacillus* (37.7%) and *Vibrio* (28.1%) in aquaculture system, which were similar with that in aquacultured yellowtail (Ramírez and Romero, 2017). However, the relative abundances of these phyla in sediment and water of aquaculture had significant differences. Firmicutes (91.6%) in sediment and Proteobacteria (71.6%) in water accounted for the highest proportion, respectively. Reportedly, some cultivable members of these phyla from aquaculture systems belonged to antibiotic resistance bacteria with (fluoro)quinolone-resistant, sulfamethoxazole-resistant, and oxytetracycline-resistant and were most abundant in fish ponds (Akinbowale et al., 2010; Hoa et al., 2011; Takasu et al., 2011; Wenguang et al., 2015). Firmicutes was described as the most predominant in the intestinal content of aquacultured animals (Ramírez and Romero, 2017). Some researchers reported that the dominant phyla of aquaculture system in Guangdong, China, were Proteobacteria, Bacteroidetes, and Firmicutes in sediment and Proteobacteria, Actinobacteria, and Bacteroidetes in water (Wenguang et al., 2015). Although only less than 1% bacteria of the microbial community could be detected by culture-dependent approach, the living bacteria can be obtained (Ramírez and Romero, 2017), which is an essential method for various researches. We only focused on culturable protease-producing bacterial community structure of tropical aquaculture system in this study; it still indirectly implied significant differences in bacterial community structure between tropical aquaculture system and other regional aquaculture systems. Reportedly, the microbiome of aquaculture systems could be more similar to structure of aquatic animal microbiomes (Ramírez and Romero, 2017).

Additionally, the composition and relative abundance of protease-producing bacteria at the genus level in sediment and water also displayed significantly different in aquaculture systems. Reportedly, *Bacillus*, *Lactobacillus*, and *Enterococcus* as protease-producing bacteria positively affected the bait digestibility and growth of aquaculture animals (Yanbo and Zirong, 2006; Ghosh et al., 2008; Suzer et al., 2008; Iehata et al., 2009; Askarian et al., 2011). In this study, *Bacillus* dominated by *B. hwajinpoensis* displayed high relative abundance in both sediment and aquaculture water. *Bacillus* as probiotic has been commonly selected to improve water quality by reducing organic matter, ammonia-nitrogen, and phosphorus accumulation and inhibit certain pathogenic bacteria of fishery by producing antimicrobial peptides, thereby making them more suitable candidates compared to other probiotics (Rengpipat et al., 1998; Verschuere et al., 2000; Hai, 2015; Kumari et al., 2016; Saravanan et al., 2018; Sonune and Garode, 2018; Yi et al., 2018; Kuebutornye et al., 2019; Wang et al., 2019). SW-72^T^ from a tidal flat of the Yellow Sea in Korea was the first reported *B. hwajinpoensis* (Yoon et al., 2003). The function of heterotrophic nitrification-aerobic denitrification of *B. hwajinpoensis* has been reported (Cheng et al., 2016), which can effectively improve water quality by removing inorganic nitrogen and total nitrogen.

The unique genera of sediment or water were also isolated in this work. Twelve unique genera were only isolated from sediment samples, *Brachybacterium*, *Chryseomicrobium*, *Curtobacterium*, *Erythrobacter*, *Halomonas*, *Isoptericola*, *Kocuria*, *Marinobacter*, *Planococcus*, *Psychrobacter*, *Rhizobium*, and *Thalassobacillus*. Meanwhile, there were seven unique genera from water samples, *Alteromonas*, *Micrococcus*, *Ornithinibacillus*, *Pseudoalteromonas*, *Pseudomonas*, *Salinicola*, and *Stenotrophomonas*. Although these unique genera accounted for tiny percentage in sediment or water, we reason that they played important roles in the stability of community structure. For example, *Brachybacterium* from *Lates calcarifer* showed inhibitory activities against *Lysinibacillus*, *Paenibacillus*, *Pseudomonas*, *Escherichia coli*, and *Mesorhizobium* (Orsod et al., 2012); *Halomonas* could improve the survival, growth, water quality, and robustness and modifies the gut microbial composition of shrimp (Gao et al., 2019); *Kocuria* as probiotic from the intestinal microbiota of rainbow trout showed resistance to sulphatriad and had health benefits in aquaculture (Sharifuzzaman et al., 2018); *Marinobacter* from a recycling aquaculture system could perform only aerobic denitrification but not nitrification (Liu et al., 2016); *Pseudomonas* showed antibiofilm properties and reduced the risk of pathogenic infection to aquaculture animals by their exopolysaccharides (Ang et al., 2020).

It is worth to note that *Vibrio* as the dominant genus was isolated from most aquaculture ponds (Figure 2 and Supplementary Table S2), which were dominated by *V. owensii* species (80 strains). The DY05^T^ and 47,666-1 belonging to the *Harveyi* clade of the genus were the first reported *V. owensii* strains from diseased cultured crustaceans (Cano-Gomez et al., 2010). So far, most of *Vibrio* from aquaculture systems in southern China belonged to *V. harveyi*, *V. alginolyticus*, *V. parahaemolyticus*, *V. splendidus*, and *V. fischeri*, whereas no *V. owensii* was detected. In this study, *V. owensii* as dominate species in tropical aquaculture systems could be isolated from most sampling stations, indicating that *V. owensii* in tropical region may be a normal species and played important roles in material circulation. Reportedly, *V. owensii* were potential bacterial pathogens in mariculture systems (Yu et al., 2013; Liu et al., 2018), necessitating the designation of an appropriate typing approach to completely fathom their transmission tactics and control infection strategies. So far, there were many reports on the molecular types of *V. alginolyticus* (Kahla-Nakbi et al., 2006), *V. tapetis* (Rodríguez et al., 2006), and *V. parahaemolyticus* (Chen et al., 2012), but no reports on that of *V. owensii*. ERIC-PCR as an easy and cost-effective genotyping approach for discriminating different strains types (Bakhshi et al., 2018) has urged us to use this technique to inspect phylogenetic closeness of *V. owensii* isolates. All 80*V. owensii* isolates produced banding patterns after amplification by ERIC-PCR (Supplementary Figure S2), indicating the complete type ability of *V. owensii* using this technology, which were clustered into four clusters (ET-1–ET-4) at 58% of similarity (Figure 4). We found that all *V. owensii* had a common band around 7,000bp (Supplementary Figure S2), indicating that they had a similar genetic background. We reason that the common band may be an effective molecular marker for *V. owensii*. The predominant clusters, ET-3 and ET-4, contained most isolates, indicating that these two genotypes were representative strains in tropical aquaculture. Additionally, the *V. owensii* strains belonging to ET-3 and ET-4 were detected in most ponds without outbreak of epidemics in this region (Supplementary Table S1) and significantly different from *V. owensii* DY05^T^ from other regions on the basis of 16S rDNA (Supplementary Figure S3), implying that *V. owensii* strains in this area may be potential beneficial bacteria for the wastewater purification and the construction of an environment-friendly aquaculture model in tropical region, especially in Hainan Island.

**Figure 5:**
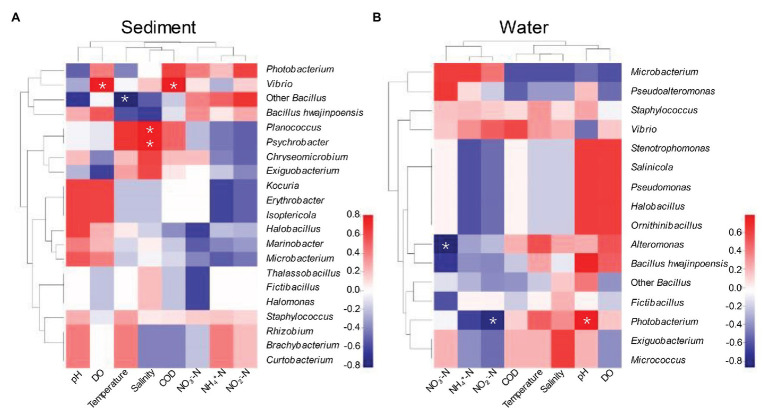
Spearman rank correlation coefficient between relative abundance of bacterial communities in sediment **(A)** and aquaculture water **(B)** and environmental variables at the genus. ^*^p < 0.05 indicates significant correlation level.

Our experimental design aimed to determine the relative influence of environmental variables of aquaculture on protease-producing microbial communities in aquaculture system. Environmental variables must play important roles in shaping protease-producing bacterial diversity and community structure in aquaculture systems (Holmström and Kjelleberg, 1999; Li et al., 2018). Therefore, Spearman rank correlation coefficient between relative abundance of protease-producing bacterial communities and environmental variables at the genus level was investigated (Figure 5). As shown in Figure 5A, DO and COD were the main factors positively affecting the distributions of dominant *Vibrio* in sediment, and salinity as a main factor also positively affected the distributions of unique genera of sediment, *Planococcus* and *Psychrobacter*. *Planococcus* spp. were associated with biological sulfamethoxazole degradation pathway, which can respond more quickly and adapt to environmental changes in aquaculture system (Kong et al., 2020). It is worth mentioning that the antagonistic effect against pathogenic species of *Psychrobacter* has been demonstrated and reported (Sun et al., 2009), and *Psychrobacter* could be commonly found in intestinal microbiota of several fish (Ringo et al., 2006; Sun et al., 2009; Yang et al., 2011; Ramirez and Romero, 2017). Reportedly, *Psychrobacter* could also improve immunological parameters and growth performance in tilapia (Makled et al., 2017), whereas temperature was the main factor negatively affecting the distribution of dominant *Bacillus* (except *B. hwajinpoensis*) in sediment, which was agreed with some previous studies in different regional oceans (Pomeroy and Wiebe, 2001; Fuhrman et al., 2008; Li et al., 2018). In this study, DO, COD, salinity, and temperature played important roles in shaping bacterial diversity of aquaculture sediment by affecting the key species. The conditions with low concentration of NO_3_^−^-N and NO_2_^−^-N were more suitable for the growth of *Alteromonas* and *Photobacterium*, and pH as a main factor positively affecting the distribution of *Photobacterium* in water (Figure 5B). *Photobacterium* genus was detected worldwide in aquaculture systems and identified as pathogens causing wound infections and hemorrhagic septicemia (Terceti et al., 2018; Labella et al., 2020). It is worth noticing that *Alteromonas* was only isolated from aquaculture water, which was identified as probiotic bacteria in aquaculture system (Kesarcodi-Watson et al., 2010; Torres et al., 2016). In conclusion, DO, temperature, salinity, and COD as the main factor could affect the protease-producing bacterial community structure of sediment, whereas pH, NO_2_^—^N, and NO_3_^−^-N were the main factors affecting that of aquaculture water.

## Conclusion

In this study, cultivable protease-producing bacterial community structures in sediment and water of tropical aquaculture systems were completely surveyed. The protease-producing bacteria displayed high diversity and complex community structure. *Bacillus* genera in sediment and *Vibrio* in aquaculture water were the dominant genera. *V. owensii* strains as the dominant *Vibrio* species were clustered into four clusters, which was identified as a novel branch of *V. owensii*. Additionally, temperature, DO, COD, salinity, NO_3_^−^-N, NO_2_^—^N, and pH of aquaculture system played important roles in shaping protease-producing bacterial diversity and community structure in aquaculture system. These results will help the development useful and practical strategies for the improvement of water quality in the marine recirculating aquaculture system.

## Data Availability Statement

The original contributions presented in the study are included in the article/Supplementary Material, further inquiries can be directed to the corresponding authors.

## Author Contributions

YW, JB, WR, and ZX designed the study and wrote the manuscript. YW, JB, HL, XZ, XC, and AH performed the experiments. YW, WR, and ZX analyzed the data. YW and JB contributed equally to this work. All authors contributed to the article and approved the submitted version.

### Conflict of Interest

The authors declare that the research was conducted in the absence of any commercial or financial relationships that could be construed as a potential conflict of interest.

## Footnotes

^1^http://eztaxon-e.ezbiocloud.net
