# Supplementary material for: Community Structure of Protease-Producing Bacteria Cultivated From Aquaculture Systems: Potential Impact of a Tropical Environment
Source: Front Microbiol. 2021 Feb 4;12:638129. doi: 10.3389/fmicb.2021.638129 (PMC7889957; doi:10.3389/fmicb.2021.638129)
Supplement: Supplementary file 1 [file Data_Sheet_1.docx]

**Supplementary Material**

Community structure of protease-producing bacteria cultivated from aquaculture systems: Potential impact of a tropical environment

Yali Wei^1, 2, a^, Jun Bu^1, 3, 4, a^, Hao Long^1, 3, 4^, Xiang Zhang^1, 3, 4^, Xiaoni Cai^1, 3, 4^, Aiyou Huang^1, 3, 4^, Wei Ren^1, 3, 4*^, Zhenyu Xie^1, 3, 4*^

^1^ State Key Laboratory of Marine Resource Utilization in the South China Sea, Hainan University, Haikou, 570228, Hainan Province, China.

^2^ Ministry of Education Key Laboratory of Cell Activities and Stress Adaptations, School of Life Sciences, Lanzhou University, Lanzhou, China.

^3^ Hainan Provincial Key Laboratory for Tropical Hydrobiology and Biotechnology, Hainan University, Haikou, 570228, Hainan Province, China.

^4^ College of Marine Sciences, Hainan University, Haikou, 570228, Hainan Province, China.

^a^ The first two authors contributed equally to this work.

*** Correspondence:**Corresponding Author
renweifly@126.com (Wei Ren); [xiezyscuta@163.com](mailto:xiezyscuta@163.com) (Zhenyu Xie)

**Figure S1.** Location of the study area (19°27′28-32″N, 110°45′13″E). The aquaculture ponds are coded 1#-6#, and Nsp represents nature seawater pool. The location is showed in Google Earth Web (https://earth.google.com/).

Table S1 50-day-stage *L. vannamei* ponds.

| Ponds | 1# | 2# | 3# | 4# | 5# | 6# |
| --- | --- | --- | --- | --- | --- | --- |
| Size (m^2^) | 2,000 | 2,000 | 2,000 | 2,000 | 2,000 | 666.67 |
| Water depth (m) | 1.5 | 1.5 | 1.5 | 1.5 | 1.5 | 1.5 |
| Healthy condition of *L. vannamei* | Healthy | Healthy | Healthy | Healthy | Healthy | Healthy |
| Aerator | 2 | 2 | 2 | 2 | 2 | 2 |
| Feed intake (kg/d) | 6 | 6 | 6 | 6 | 6 | 6 |


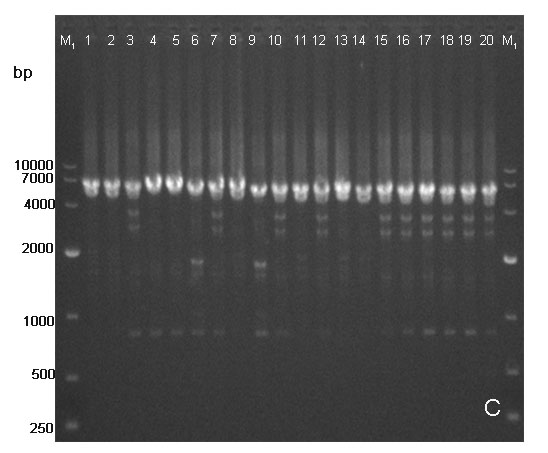

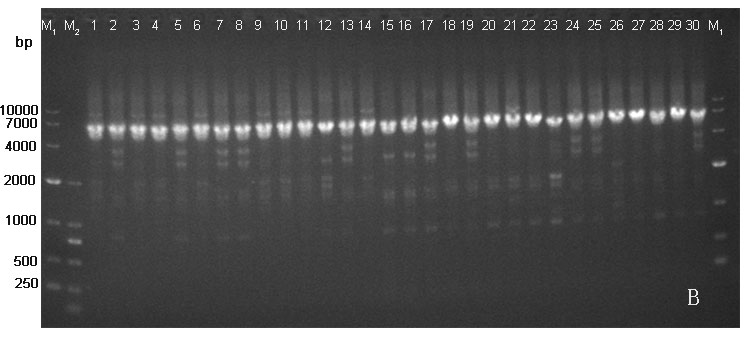

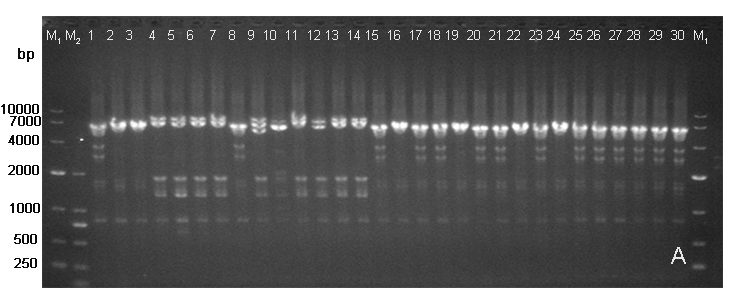
**Figure S2.** ERIC-PCR fingerprints of the *V. owensii*. M_1_-1kb DNA ladder and M_2_-DL2000 marker (Takara, Japan). **(A)** 1-S143; 2-S145; 3-S63; 4-S159; 5-S163; 6-S174; 7-S178; 8-S182; 9-S185; 10-S187; 11-S189; 12-S190; 13-S191; 14-S192; 15-S193; 16-S205; 17-S214; 18-S216; 19-S218; 20-S225; 21-S227; 22-S231; 23-S245; 24-S248; 25-S260; 26-S264; 27-S277; 28-S284; 29-S289; 30-S293. **(B)** 1-S297; 2-S300; 3-S304; 4-S306; 5-S308; 6-S312; 7-S314; 8-S315; 9-S316; 10-S319; 11-S320; 12-S321; 13-S323; 14-S329; 15-S333; 16-S339; 17-S340; 18-S342; 19-S347; 20-S350; 21-S357; 22-S360; 23-S361; 24-S362; 25-S371; 26-S372; 27-S377; 28-S381; 29-S383; 30-S392. **(C)** 1-S393; 2-S394; 3-S400; 4-S413; 5-S414; 6-S421; 7-S427; 8-S428; 9-S429; 10-432; 11-435; 12-S436; 13-S438; 14-S439; 15-S443; 16-S497; 17-S542; 18-S728; 19-S765; 20-S767.

**Table S2** Distribution of 80 *V owensii* isolates in six prawn ponds.

| Ponds | Total | Name of *V. owensii* | | | | | | |
| --- | --- | --- | --- | --- | --- | --- | --- | --- |
| 1^#^ | 3 | S143 | S145 | S63 |  |  |  |  |
| 2^#^ | 27 | S159 | S163 | S174 | S178 | S182 | S185 | S187 |
|  |  | S189 | S190 | S191 | S192 | S193 | S205 | S214 |
|  |  | S216 | S218 | S225 | 227 | S231 | S245 | S248 |
|  |  | S260 | S264 | S277 | S284 | S289 | S293 |  |
| 3^#^ | 45 | S297 | S300 | S304 | S306 | S308 | S312 | S314 |
|  |  | S315 | S316 | S319 | S320 | S321 | S323 | S329 |
|  |  | S333 | S339 | S340 | S342 | S347 | S350 | S357 |
|  |  | S360 | S361 | S362 | S371 | S372 | S377 | S381 |
|  |  | S383 | S392 | S393 | S394 | S400 | S413 | S414 |
|  |  | S421 | S427 | S428 | S429 | S432 | S435 | S436 |
|  |  | S438 | S439 | S443 |  |  |  |  |
| 4^#^ | 1 | S497 |  |  |  |  |  |  |
| 5^#^ | 1 | S542 |  |  |  |  |  |  |
| 6^#^ | 3 | S728 | S765 | S767 |  |  |  |  |

**Table S3** Distribution of 80 *V owensii* isolates in four ERIC-PCR fingerprint clusters.

| Cluster | Total | Name of *V. owensii* | | | | | | |
| --- | --- | --- | --- | --- | --- | --- | --- | --- |
| ET-1 | 3 | S321 | S421 | S429 |  |  |  |  |
| ET-2 | 9 | S159 | S163 | S174 | S178 | S185 | S189 | S190 |
|  |  | S191 | S192 |  |  |  |  |  |
| ET-3 | 32 | S63 | S145 | S187 | S205 | S218 | S231 | S248 |
|  |  | S297 | S304 | S306 | S312 | S316 | S319 | S320 |
|  |  | S329 | S342 | S350 | S357 | S360 | S361 | S372 |
|  |  | S377 | S381 | S383 | S393 | S394 | S413 | S414 |
|  |  | S428 | S435 | S438 | S439 |  |  |  |
| ET-4 | 36 | S182 | S143 | S214 | S216 | S225 | S227 | S245 |
|  |  | S193 | S260 | S264 | S277 | S284 | S289 | S293 |
|  |  | S300 | S308 | S314 | S315 | S323 | S333 | S339 |
|  |  | S392 | S400 | S427 | S432 | S436 | S443 | S340 |
|  |  | S347 | S362 | S371 | S497 | S728 | S765 | S767 |
|  |  | S542 |  |  |  |  |  |  |


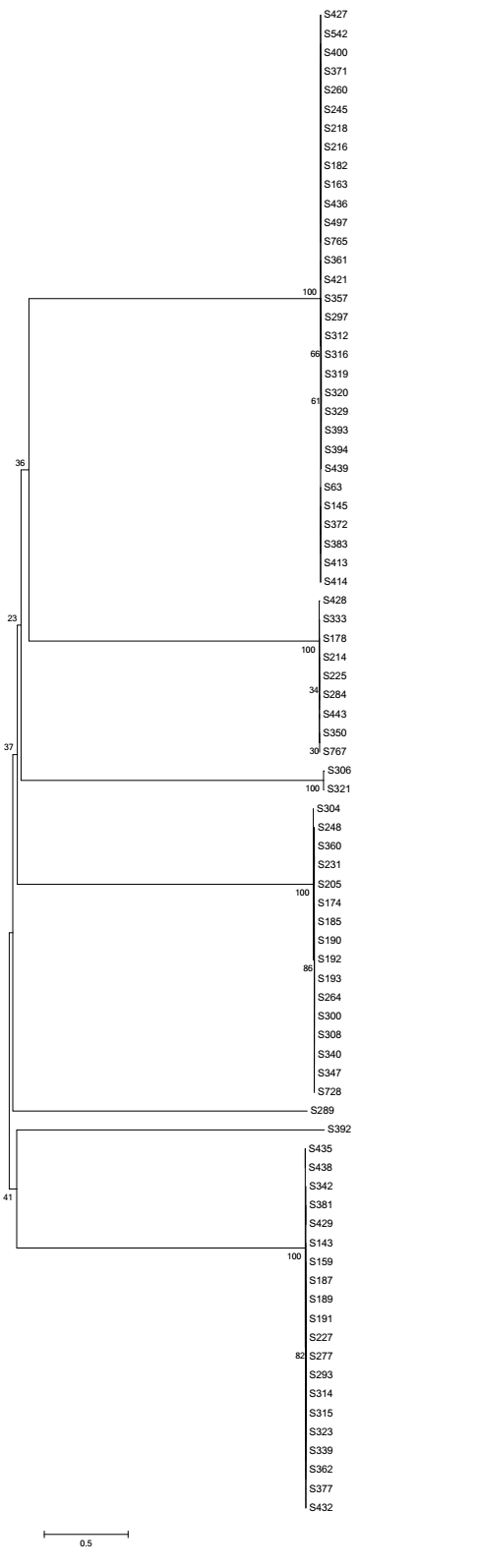


**Figure S3.** Phylogenetic tree analysis of 16S rDNA gene sequences of 80 *V. owensii* strains.
